# Supplementary material for: Growth tradeoffs produce complex microbial communities on a single limiting resource
Source: Nat Commun. 2018 Aug 10;9:3214. doi: 10.1038/s41467-018-05703-6 (PMC6086922; doi:10.1038/s41467-018-05703-6)
Supplement: Supplementary file 1 — Supplementary Information [file 41467_2018_5703_MOESM1_ESM.pdf]

Supplementary Information: Growth tradeoffs produce complex microbial communities on a single limiting resource

Manhart and Shakhnovich

SUPPLEMENTARY NOTE 1. DERIVATION OF THE SELECTION COEFFICIENTS

Here we derive Eqs. 2 and 3, which show how the selection coefficients s_{ij} (Eq. 1) depend on the underlying parameters. We assume the nontrivial case in which the saturation time is longer than each strain's lag time ($t_{\text{sat}} > \max_k \lambda_k$). Using the minimal growth model in Eq. 8, the selection coefficient definition in Eq. 1 simplifies to

$$s_{ij} = \frac{1}{\tau_i}(t_{\text{sat}} - \lambda_i) - \frac{1}{\tau_j}(t_{\text{sat}} - \lambda_j). \tag{1}$$

We next rewrite the saturation condition (Eq. 10) in terms of the selection coefficients relative to strain i:

$$1 = e^{(t_{\text{sat}} - \lambda_i)/\tau_i} \left(\sum_k \frac{x_k}{\rho Y_k} e^{s_{ki}} \right), \tag{2}$$

where we have inserted the initial density $x_k = N_k(0)/\sum_\ell N_\ell(0)$ for each strain k and the initial resource density per cell $\rho = R/\sum_\ell N_\ell(0)$. We can then solve for t_{sat} and expand to first order in each s_{ki}:

$$\begin{aligned} t_{\text{sat}} &= \lambda_i - \tau_i \log \left(\sum_k \frac{x_k}{\rho Y_k} e^{s_{ki}} \right) \\ &\approx \lambda_i + \tau_i \left(\log\left[\rho \bar{Y}\right] - \bar{Y} \sum_k \frac{x_k}{Y_k} s_{ki} \right), \end{aligned} \tag{3}$$

where $\bar{Y} = \left(\sum_k x_k/Y_k\right)^{-1}$ is the harmonic mean of the yields (Eq. 4). However, since we can freely choose a different strain j to be the reference strain, we must also have

$$t_{\text{sat}} \approx \lambda_j + \tau_j \left(\log\left[\rho \bar{Y}\right] - \bar{Y} \sum_k \frac{x_k}{Y_k} s_{kj} \right). \tag{4}$$

To be self-consistent these two expressions for t_{sat} must be equal for any i and j, which leads to the following system of linear equations for the selection coefficients:

$$\begin{aligned} s_{ij} - \bar{Y} \frac{\Delta\tau_{ij}}{\tau_i} \sum_k \frac{x_k}{Y_k} s_{kj} &= \\ &- \frac{1}{\tau_i} \left(\Delta\tau_{ij} \log\left[\rho \bar{Y}\right] + \Delta\lambda_{ij} \right), \end{aligned} \tag{5}$$

where $\Delta\lambda_{ij} = \lambda_i - \lambda_j$ and $\Delta\tau_{ij} = \tau_i - \tau_j$.

We now take the solution for s_{ij} in Eqs. 2 and 3 as an ansatz and show that it satisfies this system of equations. If we substitute this expression for s_{ij} and s_{kj} in Supplementary Eq. 5, the left-hand side (LHS) of the system in Supplementary Eq. 5 becomes

$$\begin{aligned} \text{LHS} = &-\frac{\bar{\tau}}{\tau_i \tau_j} \left(\Delta\tau_{ij} \log\left[\rho \bar{Y}\right] + \Delta\lambda_{ij} + \bar{Y} \sum_k \frac{x_k}{Y_k \tau_k} \left[\Delta\tau_{ik} \Delta\lambda_{kj} - \Delta\lambda_{ik} \Delta\tau_{kj} \right] \right) \\ &+ \bar{Y} \frac{\Delta\tau_{ij}}{\tau_i} \sum_k \frac{x_k}{Y_k} \frac{\bar{\tau}}{\tau_k \tau_j} \left(\Delta\tau_{kj} \log\left[\rho \bar{Y}\right] + \Delta\lambda_{kj} + \bar{Y} \sum_\ell \frac{x_\ell}{Y_\ell \tau_\ell} \left[\Delta\tau_{k\ell} \Delta\lambda_{\ell j} - \Delta\lambda_{k\ell} \Delta\tau_{\ell j} \right] \right). \end{aligned} \tag{6}$$

Since

$$\sum_k \sum_\ell \frac{x_k}{Y_k \tau_k} \frac{x_\ell}{Y_\ell \tau_\ell} \left[\Delta\tau_{k\ell} \Delta\lambda_{\ell j} - \Delta\lambda_{k\ell} \Delta\tau_{\ell j} \right] = 0 \tag{7}$$

because the summand is antisymmetric in the summation indices, we drop the inner sum over ℓ and combine the remaining sums over k to obtain

$$\begin{aligned} \text{LHS} = -\frac{\bar{\tau}}{\tau_i \tau_j} \Bigg(& \Delta\tau_{ij} \log\left[\rho \bar{Y}\right] + \Delta\lambda_{ij} + \bar{Y} \sum_k \frac{x_k}{Y_k \tau_k} \\ & \times \left[\Delta\tau_{ik} \Delta\lambda_{kj} - \Delta\lambda_{ik} \Delta\tau_{kj} \right. \\ & \left. - \Delta\tau_{ij} \Delta\tau_{kj} \log\left(\rho \bar{Y}\right) - \Delta\tau_{ij} \Delta\lambda_{kj} \right] \Bigg). \end{aligned} \tag{8}$$

We cancel out terms and factor to obtain

$$\begin{aligned}\text{LHS} &= -\frac{\bar{\tau}}{\tau_i\tau_j}(\Delta\tau_{ij}\log\left[\rho\bar{Y}\right]+\Delta\lambda_{ij})\left(1-\bar{Y}\sum_k\frac{x_k}{Y_k\tau_k}\Delta\tau_{kj}\right)\\ &= -\frac{\bar{\tau}}{\tau_i\tau_j}(\Delta\tau_{ij}\log\left[\rho\bar{Y}\right]+\Delta\lambda_{ij})\left(1-\left[1-\frac{\tau_j}{\bar{\tau}}\right]\right)\\ &= -\frac{1}{\tau_i}(\Delta\tau_{ij}\log\left[\rho\bar{Y}\right]+\Delta\lambda_{ij}),\end{aligned} \tag{9}$$

where we have used the definitions in Eq. 4 to invoke

$$\bar{Y}\sum_k\frac{x_k}{\tau_k Y_k}\Delta\tau_{kj}=1-\frac{\tau_j}{\bar{\tau}}. \tag{10}$$

This equals the right side of Supplementary Eq. 5, proving the solution is correct.

SUPPLEMENTARY NOTE 2. SATURATION TIME AND OVERALL YIELD FOR A MIXED POPULATION

Here we calculate expressions for the saturation time and overall yield for a mixed population of multiple strains, which provide interpretations of the quantities $\bar{\tau}$ and $\bar{Y}$ (Eq. 4) as the effective growth time and effective yield of the whole population. Using the approximation for t_{sat} in Supplementary Eq. 3, which holds for any reference strain i, we insert the selection coefficients s_{ki} from the expression in Eqs. 2 and 3:

$$\begin{aligned}t_{\text{sat}} &\approx \lambda_i+\tau_i\left(\log\left[\rho\bar{Y}\right]-\bar{Y}\sum_k\frac{x_k}{Y_k}s_{ki}\right)\\ &= \lambda_i+\tau_i\log\left(\rho\bar{Y}\right)+\tau_i\bar{Y}\sum_k\frac{x_k}{Y_k}\frac{\bar{\tau}}{\tau_k\tau_i}\left(\Delta\tau_{ki}\log\left[\rho\bar{Y}\right]+\Delta\lambda_{ki}+\bar{Y}\sum_\ell\frac{x_\ell}{Y_\ell\tau_\ell}\left[\Delta\tau_{k\ell}\Delta\lambda_{\ell i}-\Delta\lambda_{k\ell}\Delta\tau_{\ell i}\right]\right).\end{aligned} \tag{11}$$

We eliminate the double sums over k and ℓ (using Supplementary Eq. 7) and invoke the identity in Supplementary Eq. 10 to obtain

$$t_{\text{sat}}\approx\sum_k x_k\lambda_k\frac{\bar{\tau}\bar{Y}}{\tau_k Y_k}+\bar{\tau}\log\left(\rho\bar{Y}\right). \tag{12}$$

Comparing with the saturation time for a homogeneous population of a single strain (Eq. 9), we see the weighted sum over all lag times in Supplementary Eq. 12 corresponds to the effective time shift from the lag phase, while the last term in Supplementary Eq. 12 determines the time during which exponential growth occurs. In analogy with Eq. 9, $\bar{\tau}$ is therefore the mixed population's effective exponential growth time (reciprocal growth rate), and $\bar{Y}$ is the mixed population's effective yield. Note that the effective growth rate $1/\bar{\tau}$ is just an arithmetic mean of the individual strains' growth rates.

The quantity $\bar{Y}$ is in fact the exact yield for a mixed population when all strains are neutral. Let $N_{\text{sat}}=\sum_k N_k(t_{\text{sat}})$ be the total population size at saturation. For a set of strains to all be neutral, they must have a fixed lag-growth tradeoff $c_{ij}=-\Delta\lambda_{ij}/\Delta\tau_{ij}=c$ for all pairs of strains i and j (Eq. 6, Supplementary Note 3). In that case the effective yield is $\bar{Y}=e^c/\rho$. Since all $s_{ij}=0$ by definition, then Supplementary Eq. 3 implies that $t_{\text{sat}}=\lambda_k+c\tau_k$ for any strain k, which we can rewrite as $(t_{\text{sat}}-\lambda_k)/\tau_k=c$. We then calculate the total population size at saturation to be

$$\begin{aligned}N_{\text{sat}} &= \sum_k N_k(0)e^{(t_{\text{sat}}-\lambda_k)/\tau_k}\\ &= e^c\sum_k N_k(0)\\ &= \rho\bar{Y}\sum_k N_k(0)\\ &= R\bar{Y},\end{aligned} \tag{13}$$

where we have used the definition $\rho=R/\sum_k N_k(0)$. This shows that the total saturation size N_{sat} is proportional to the total amount of resources R, with proportionality constant $\bar{Y}$, meaning that $\bar{Y}$ is indeed the yield for the whole population.

SUPPLEMENTARY NOTE 3. CONDITIONS FOR NEUTRAL COEXISTENCE AND MULTISTABILITY

Here we derive the conditions that lead to neutral coexistence or multistability in population dynamics. Both phenomena require the existence of nontrivial fixed points in the space of densities, where all pairwise selection coefficients are zero. We first determine the conditions on parameters necessary for these fixed points to exist.

Conditions for fixed points

We substitute $s_{ij} = 0$ into the linear system for the selection coefficients (Supplementary Eq. 5), which implies the right-hand side of the system must be zero: $\Delta\tau_{ij} \log\left(\rho\bar{Y}\right) + \Delta\lambda_{ij} = 0$, or equivalently $\rho\bar{Y} = e^{-\Delta\lambda_{ij}/\Delta\tau_{ij}}$. Since this must hold for all pairs of strains i and j, $\Delta\lambda_{ij}/\Delta\tau_{ij}$ is therefore a constant independent of i and j:

$$c = -\frac{\Delta\lambda_{ij}}{\Delta\tau_{ij}}. \tag{14}$$

This is the first requirement for a fixed point (equivalent to the linear lag-growth tradeoff condition in Eq. 6). The second requirement is therefore

$$\rho\bar{Y} = e^{c}. \tag{15}$$

This equation implies that $c > 0$ (lag and growth must have a tradeoff, rather than a synergy, across strains), since the left-hand side of Supplementary Eq. 15 is the fold-change of the whole population's growth and therefore must be greater than 1. Supplementary Equation 15 furthermore imposes a constraint on the initial density of resources ρ. Since $\bar{Y}$ is the harmonic mean of the yields $\{Y_k\}$ (Eq. 4), it is bounded by the minimum and maximum yields across strains:

$$\min_k Y_k < \bar{Y} < \max_k Y_k. \tag{16}$$

Combining this constraint with Supplementary Eq. 15, we obtain the limits on the resource density ρ in Eq. 7.

Space of fixed-point densities

The condition in Supplementary Eq. 15 also imposes constraints on the strain densities that can be fixed points. Substituting in the definition of $\bar{Y}$ to Supplementary Eq. 15, a set of densities $\{\tilde{x}_k\}$ at which the strains have a fixed point must satisfy the following linear equation:

$$\sum_k \frac{\tilde{x}_k}{Y_k} = \rho e^{-c}. \tag{17}$$

If there are M total strains, then the space of fixed points is a section of an $(M-2)$-dimensional hyperplane, since the M densities must satisfy two linear equations (Supplementary Eq. 17 as well as normalization $\sum_k \tilde{x}_k = 1$). Supplementary Fig. 1 shows these strain densities for $M = 3$ (Supplementary Fig. 1a) and $M = 4$ strains (Supplementary Fig. 1b) as functions of the resource density ρ, which determines the relative proportion of each strain at the fixed points. In general, smaller ρ leads to fixed points with a greater density of high-yield strains, while larger ρ leads to fixed points with more low-yield strains.

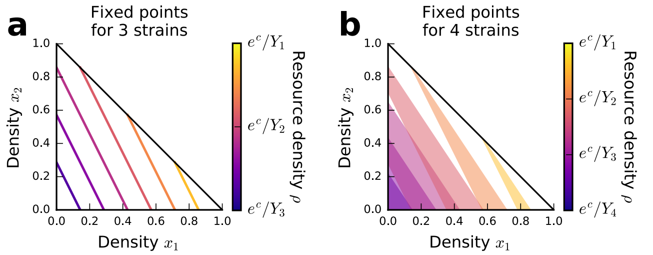

SUPPLEMENTARY FIGURE 1. **Space of fixed-point densities.** (**a**) For three strains, the space of fixed-point densities (satisfying Supplementary Eq. 17) is one-dimensional. We project this one-dimensional region into the space of densities for two strains x_1 and x_2. Each line corresponds to fixed points for a different value of the resource density ρ (indicated by the color). (**b**) Same as (**a**) but for four strains, where the space of fixed-point densities is two-dimensional. See Supplementary Note 8 for parameter values.

Stability of density fluctuations at fixed points: neutral coexistence or multistability

Density fluctuations will occur from both extrinsic and intrinsic noise, such as the random sampling of the population from one round of competition to the next [1]. Let $\tilde{\mathbf{x}} = \{\tilde{x}_k\}$ be a set of densities satisfying the fixed-point condition (Supplementary Eq. 17). To determine the stability, we consider small fluctuations $\Delta\mathbf{x}$ around this point. Let the Jacobian of the selection coefficients at the fixed point be

$$\mathcal{J}_{ijk} = \left.\frac{\partial s_{ij}}{\partial x_k}\right|_{\mathbf{x}=\tilde{\mathbf{x}}} \tag{18}$$

so that

$$s_{ij}(\tilde{\mathbf{x}} + \Delta\mathbf{x}) \approx \sum_k \mathcal{J}_{ijk}\Delta x_k. \tag{19}$$

For small fluctuations we can approximate the density dynamics around the fixed point with the differential equations in Eq. 12:

$$\begin{aligned}\frac{d}{dr}\Delta x_i &= (\tilde{x}_i + \Delta x_i)\sum_k(\tilde{x}_k + \Delta x_k)s_{ik}(\tilde{\mathbf{x}} + \Delta\mathbf{x}) \\ &\approx \tilde{x}_i \sum_k \tilde{x}_k \sum_j \mathcal{J}_{ikj}\Delta x_j \\ &= \sum_j W_{ij}\Delta x_j,\end{aligned} \tag{20}$$

where we have dropped higher-order terms in $\Delta\mathbf{x}$ and defined the matrix

$$W_{ij} = \tilde{x}_i \sum_k \tilde{x}_k \mathcal{J}_{ikj}. \tag{21}$$

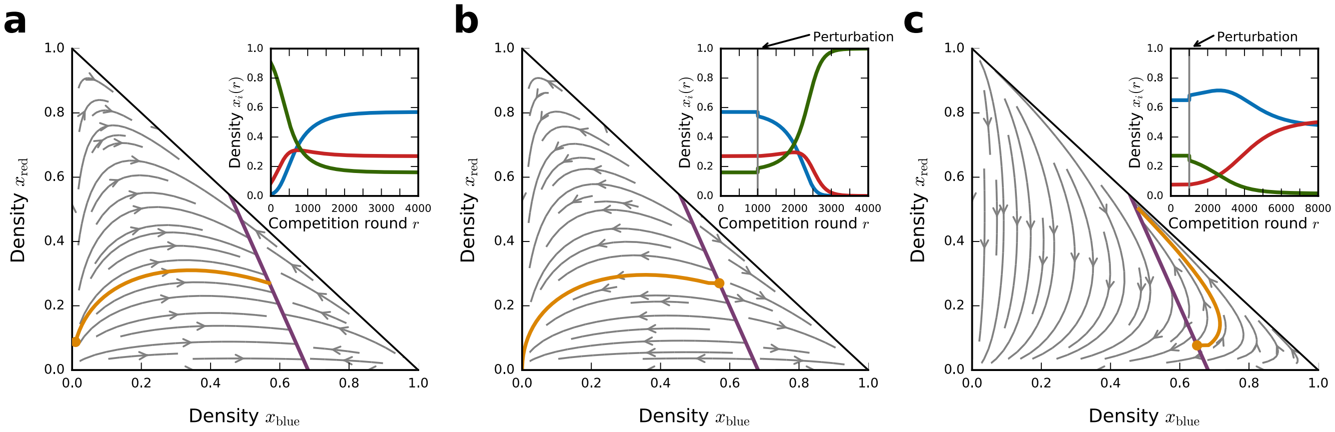

SUPPLEMENTARY FIGURE 2. **Phase portraits of neutral coexistence and multistability.** Dynamics of three strains (blue, red, green) projected into the space of densities x_{blue} and x_{red}. Gray streamlines show dx_i/dr (Eq. 12), while the magenta line indicates the set of fixed points (Supplementary Eq. 17). The orange curve is an example trajectory beginning at the orange dot. Insets show the density $x_i(r)$ over competition rounds r for each strain along the orange trajectory. (**a**) Case where all fixed points are stable to fluctuations off the space of fixed points; (**b**) case where all fixed points are unstable; (**c**) case where there is a mix of stable and unstable fixed points. The vertical gray lines in the insets of panels (**b**) and (**c**) indicate the time at which the densities are perturbed away from unstable fixed points. See Supplementary Note 8 for parameter values.

To analyze stability of fluctuations around the fixed point, we must determine the eigenvalues of the matrix W_{ij}: negative eigenvalues will correspond to directions in the space of densities that are stable to small fluctuations, positive eigenvalues will indicate unstable directions, and zero eigenvalues indicate neutral directions. We calculate the Jacobian of the selection coefficient using the formula in Eqs. 2 and 3. We first note that the density derivatives of $\bar{\tau}$ and $\bar{Y}$ are

$$\frac{\partial \bar{\tau}}{\partial x_k} = \bar{\tau}\frac{\bar{Y}}{Y_k}\left(1 - \frac{\bar{\tau}}{\tau_k}\right), \qquad \frac{\partial \bar{Y}}{\partial x_k} = -\frac{\bar{Y}^2}{Y_k}. \tag{22}$$

Therefore the derivatives of the selection coefficient are

$$\begin{aligned}
\frac{\partial}{\partial x_k} s_{ij}^{\text{lag}} &= -\frac{\bar{\tau}\Delta\lambda_{ij}}{\tau_i\tau_j}\frac{\bar{Y}}{Y_k}\left(1 - \frac{\bar{\tau}}{\tau_k}\right), \\
\frac{\partial}{\partial x_k} s_{ij}^{\text{growth}} &= -\frac{\bar{\tau}\Delta\tau_{ij}}{\tau_i\tau_j}\frac{\bar{Y}}{Y_k}\left(\left[1 - \frac{\bar{\tau}}{\tau_k}\right]\log\left[\rho\bar{Y}\right] - 1\right), \\
\frac{\partial}{\partial x_k} s_{ij\ell}^{\text{coupling}} &= -\frac{\bar{\tau}\bar{Y}}{\tau_\ell Y_\ell}\left(\frac{\Delta\tau_{i\ell}\Delta\lambda_{\ell j} - \Delta\lambda_{i\ell}\Delta\tau_{\ell j}}{\tau_i\tau_j}\right) \\
&\quad \times \left(\delta_{\ell k} - x_\ell \frac{\bar{\tau}\bar{Y}}{\tau_k Y_k}\right).
\end{aligned} \tag{23}$$

Combining these components and evaluating them at a fixed point (Supplementary Eqs. 14 and 15) results in

$$\mathcal{J}_{ijk} = \frac{\bar{\tau}\Delta\tau_{ij}}{\tau_i\tau_j}\frac{\bar{Y}}{Y_k}. \tag{24}$$

Therefore the matrix for dynamics around a fixed point is

$$\begin{aligned}
W_{ij} &= \tilde{x}_i \sum_k \tilde{x}_k \frac{\bar{\tau}\Delta\tau_{ik}}{\tau_i\tau_k}\frac{\bar{Y}}{Y_j} \\
&= \tilde{x}_i \frac{\bar{\tau}\bar{Y}}{\tau_i Y_j}\left(\tau_i \sum_k \frac{\tilde{x}_k}{\tau_k} - 1\right).
\end{aligned} \tag{25}$$

This matrix has outer-product form $W_{ij} = a_i b_j$. It is straightforward to show that such a matrix has an eigenvalue $\mu = \sum_i a_i b_i$, while all other eigenvalues are zero. The zero eigenvalues correspond to the neutral directions within the space of fixed points (Supplementary Fig. 1), while the one nonzero eigenvalue is the only direction orthogonal to this space. This eigenvalue is

$$\begin{aligned}
\mu &= \sum_i \tilde{x}_i \frac{\bar{\tau}\bar{Y}}{\tau_i Y_i}\left(\tau_i \sum_k \frac{\tilde{x}_k}{\tau_k} - 1\right) \\
&= \bar{\tau}\sum_k \frac{\tilde{x}_k}{\tau_k} - 1,
\end{aligned} \tag{26}$$

where we have simplified using the definitions of $\bar{\tau}$ and $\bar{Y}$ (Eq. 4).

Density fluctuations are stable in the non-neutral direction when $\mu < 0$, which we can rewrite as

$$\sum_k \frac{\tilde{x}_k}{\tau_k Y_k} - \left(\sum_k \frac{\tilde{x}_k}{\tau_k}\right)\left(\sum_k \frac{\tilde{x}_k}{Y_k}\right) > 0. \tag{27}$$

That is, fluctuations orthogonal to the space of fixed points are stable when the covariance of reciprocal

growth times and reciprocal yields is positive. If slower-growing strains always have higher yields (growth-yield tradeoff), the covariance is positive for any set of densities $\{\tilde{x}_k\}$, and so any fluctuations off the space of fixed points will be stabilized. In this case the fixed points correspond to neutral coexistence of the strains. Supplementary Fig. 2a shows an example for three strains; selection drives the population to the space of fixed points from any initial state. The population will then fluctuate randomly within this space (neutral dynamics, not shown). If slower-growing strains always have lower yields (growth-yield synergy), then the covariance is negative for any densities, and fluctuations off the fixed points will be unstable. This means the population is multistable, such that it will converge to completely different compositions of strains depending on its initial state relative to the space of fixed points. For example, Supplementary Fig. 2b shows how a small perturbation in one direction off a fixed point leads to fixation of the green strain, while a perturbation in the opposite different direction would have led to fixation of the blue strain. If no perfect correlation holds across the growth times and yields of the strains, then the sign of the covariance may depend on the densities $\{\tilde{x}_k\}$, leading to a mix of neutral coexistence and multistability as in Supplementary Fig. 2c. In that case it is possible for a population that is perturbed away from a fixed point with an unstable non-neutral direction to evolve to a different fixed point with a stable non-neutral direction.

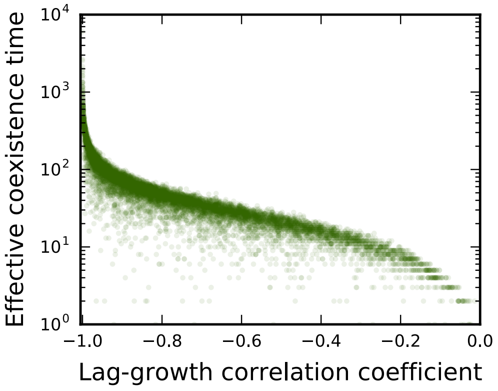

SUPPLEMENTARY FIGURE 3. **Effective coexistence of strains with approximate lag-growth tradeoffs.** Each green point represents a set of 100 strains with randomly-generated trait values. We determine the empirical lag-growth tradeoff c and correlation coefficient from the linear regression of lag and growth times. The population starts at the maximum-entropy set of densities that would allow neutral coexistence (Supplementary Eq. 29) if the strains' lag and growth times fell exactly on the regression line. We exclude realizations where ρ falls outside of the allowed range (Eq. 7) given the tradeoff c and yields $\{Y_k\}$, or if any of the maximum-entropy densities is too small ($< 10^{-6}$) to constitute meaningful coexistence. We then evolve the densities over competition rounds and determine the number of rounds until any strain goes extinct (i.e., its density falls below 10^{-6}), which we use as the time of effective coexistence. For each of the 100 communities, this time is plotted on the vertical axis against the empirical lag-growth correlation coefficient on the horizontal axis. Green points are transparent to show their density. See Supplementary Note 8 for parameter values.

Maximum entropy of strains with neutral coexistence

For strains that can neutrally coexist, the set of densities $\{\tilde{x}_k\}$ with maximum diversity over strains is of particular interest. A common way to measure strain diversity in a population is by Shannon entropy, defined as

$$S = -\sum_k \tilde{x}_k \log \tilde{x}_k. \tag{28}$$

This ranges from zero if only one strain is present, to $\log M$ if M strains are equally abundant. The condition on densities with neutral coexistence (Supplementary Eq. 17) means that the reciprocal yield averaged over densities must be ρe^{-c}. With this constraint the maximum-entropy set of densities is of Boltzmann form [2], with reciprocal yield in the role of energy:

$$\tilde{x}_k = \frac{1}{Z} e^{-\beta/Y_k}, \tag{29}$$

where β is defined such that

$$\frac{\sum_k \frac{1}{Y_k} e^{-\beta/Y_k}}{\sum_k e^{-\beta/Y_k}} = \rho e^{-c} \tag{30}$$

and $Z = \sum_k e^{-\beta/Y_k}$ is the normalization constant. The maximum entropy is therefore

$$S_{\max} = \beta \rho e^{-c} + \log\left(\sum_k e^{-\beta/Y_k}\right). \tag{31}$$

The parameter β (analogous to inverse temperature) sets a yield threshold determining how much of the population consists of strains with low yields versus those with high yields. If ρ is close to $e^c/\min_k Y_k$ (high end of range in Eq. 7), then β will be negative and large in magnitude, meaning the strains with the lowest yields will be favored. Similarly, if ρ is close to $e^c/\max_k Y_k$ (low end of range in Eq. 7), then β will be large and positive, so that strains with the highest yields will be favored. All M strains will be equally represented ($x_k = 1/M$) if $\beta = 0$, which occurs if the resource density is set to

$$\rho = \frac{e^c}{M} \sum_k \frac{1}{Y_k}. \tag{32}$$

Effective coexistence with noisy tradeoffs

The condition for neutral coexistence in Eq. 6 is an exact linear tradeoff between lag and growth times. However, a tradeoff among real strains will never be exactly linear for more than two strains. But even if the tradeoff is noisy, with some fluctuations around a linear trend, this will still lead to effective coexistence over some finite time scale, which may be sufficiently long to be biologically relevant (e.g., to observe in a laboratory experiment, or before new mutations arise or the environment changes). To illustrate this, we randomly generate communities (sets of strains with distinct growth traits) with different correlations of lag and growth times across strains. We initialize each community at a set of densities $\{x_k\}$ such that it would coexist if all the traits exactly obeyed the linear regression of growth traits. We then measure the time (number of competition rounds) it takes for the first strain to go extinct, i.e., for its density to drop below a certain threshold. Supplementary Fig. 3 shows that while communities with only weak lag-growth correlations will generally not coexist for very long, as expected, the apparent coexistence time increases rapidly as the correlation becomes stronger. Thus, a community with even moderate correlation may still practically coexist over a significant time.

SUPPLEMENTARY NOTE 4. SELECTION ON AN INVADER TO A COEXISTING COMMUNITY

Consider a strain that invades a community of neutrally-coexisting strains. We assume the invader enters at infinitesimally-low density. In this case, the quantities $\bar{\tau}$ and $\bar{Y}$ (Eq. 4) for the coexisting strains combined with the invader are essentially the same as their values for the coexisting strains alone. In particular, $\log\left(\rho\bar{Y}\right) \approx c$ (Supplementary Eq. 15), where c is the lag-growth tradeoff for the coexisting strains. The selection coefficient of the invader relative to each coexisting strain j is therefore

$$\begin{aligned}
s_{\mathrm{inv},j} &= -\frac{\bar{\tau}}{\tau_{\mathrm{inv}}\tau_j}\left(\Delta\lambda_{\mathrm{inv},j} + \Delta\tau_{\mathrm{inv},j}\log\left[\rho\bar{Y}\right] + \bar{Y}\sum_k \frac{x_k}{\tau_k Y_k}\left[\Delta\tau_{\mathrm{inv},k}\Delta\lambda_{kj} - \Delta\lambda_{\mathrm{inv},k}\Delta\tau_{kj}\right]\right) \\
&\approx -\frac{\bar{\tau}}{\tau_{\mathrm{inv}}\tau_j}\left(\Delta\lambda_{\mathrm{inv},j} + c\Delta\tau_{\mathrm{inv},j}\right)\left(1 - \frac{e^c}{\rho}\sum_{k\neq\mathrm{inv}}\frac{x_k}{\tau_k Y_k}\Delta\tau_{kj}\right) \\
&= -\frac{1}{\tau_{\mathrm{inv}}}\left(\Delta\lambda_{\mathrm{inv},j} + c\Delta\tau_{\mathrm{inv},j}\right) \\
&= -\frac{1}{\tau_{\mathrm{inv}}}\left(c\tau_{\mathrm{inv}} + \lambda_{\mathrm{inv}} - \mathrm{constant}\right),
\end{aligned} \tag{33}$$

where we have used $\lambda_j = -c\tau_j + \text{constant}$ (Eq. 6) for the coexisting strains. This shows that the selection coefficients between the invader and each of the coexisting strains j are the same: if the invader's lag and growth times are above the diagonal line of the coexisting strains (e.g., Fig. 3a), then the the invader will have negative selection coefficient relative to all coexisting strains, and so its densities will decay to zero. Otherwise, its selection coefficient will be positive and the invader will take over.

SUPPLEMENTARY NOTE 5. PAIRWISE VERSUS COMMUNITY COEXISTENCE

A collection of strains that neutrally coexist in pairs will only coexist all together if they share the same lag-growth tradeoff c (Eq. 6). In this case, though, the resource densities ρ at which each pair coexists will not be the same, nor will they be the same as the values of ρ at which the whole community coexists. Consider three strains with a lag-growth tradeoff c and in order of increasing yields, so that $e^c/Y_3 < e^c/Y_2 < e^c/Y_1$. For strains 1 and 2 to coexist, ρ must be between e^c/Y_2 and e^c/Y_1, while for strains 2 and 3 to coexist, ρ must be between e^c/Y_3 and e^c/Y_2. These constraints are mutually exclusive, so strains 1 and 2 will not coexist in the same environmental conditions as strains 2 and 3. Furthermore, all three strains can coexist as long as $e^c/Y_3 < \rho < e^c/Y_1$, but for any value of ρ in that range, one of the pairs will not coexist. Therefore some, but not all, pairs of strains from a coexisting community will coexist on their own in the same environment.

SUPPLEMENTARY NOTE 6. PAIRWISE CHAMPION MUST WIN MIXED COMPETITION WITH EQUAL YIELDS

Figure 4a,b gives an example of strains where the pairwise champion (green strain, which wins each pairwise competition) does not necessarily win the mixed com-

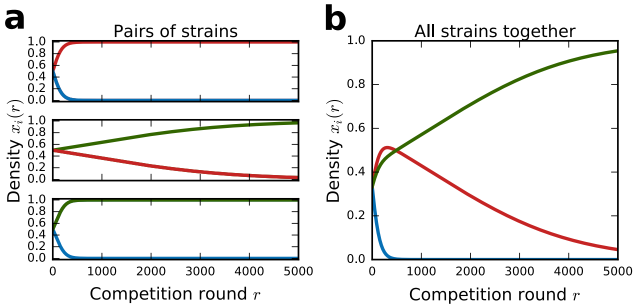

SUPPLEMENTARY FIGURE 4. **Pairwise champion always wins with equal yields.** (**a**) Density dynamics $x_i(r)$ for pairwise competitions between three strains (blue, red, green) with a single pairwise champion (green). (**b**) Density dynamics $x_i(r)$ for a competition of all three strains starting from equal densities. See Supplementary Note 8 for parameter values.

petition with all strains present. However, that outcome requires the three strains to have significantly different yields. Here we show that if the strains have equal yields, then the pairwise champion must always win the mixed competition, although it can still lose on short time scales.

Define the signed component of the selection coefficient to be

$$\sigma_{ij} = \frac{\tau_i \tau_j}{\bar{\tau}} s_{ij}. \quad (34)$$

That is, we remove the overall factor of $\bar{\tau}/(\tau_i \tau_j)$ from s_{ij} (Eqs. 2 and 3) since it is always positive and therefore does not affect the overall sign. For a set of strains with equal yields, these signed components are convenient because their values for the mixed competition $\sigma_{ij}^{\text{mixed}}$, where all strains are present, have a simple relationship to their values for pairwise competitions, $\sigma_{ij}^{\text{pair}}$, where only i and j are present:

$$\sigma_{ij}^{\text{mixed}} = (x_i + x_j)\sigma_{ij}^{\text{pair}} + \sum_{k \neq i,j} \frac{x_k}{\tau_k} \left(\tau_j \sigma_{ik}^{\text{pair}} - \tau_i \sigma_{jk}^{\text{pair}} \right). \quad (35)$$

That is, the signed component of the selection coefficient on i relative to j in the mixed competition is a linear combination of the selection coefficient from their pairwise competition, weighed by the fraction of the mixed population consisting of i and j, with the pairwise selection coefficients of i and j relative to all other strains k, each weighed by the density of that other strain.

In the case of equal yields, selection coefficients for pairwise competitions must obey transitivity [1], and therefore there must be one strain that wins all of the pairwise competitions, and another strain that loses all of them. If i is the winner of all pairwise competitions ($\sigma_{ik}^{\text{pair}} > 0$ for all k) and j the loser ($\sigma_{jk}^{\text{pair}} < 0$ for all k), then Supplementary Eq. 35 shows that $\sigma_{ij}^{\text{mixed}} > 0$, i.e., the pairwise winner must always beat the pairwise loser in the mixed competition. Therefore the loser j is guaranteed to go extinct before i can. But once j goes extinct, the same argument holds for the next-worst strain among the remaining ones, so that it, too, must go extinct before i. Eventually i will be left with just one other strain, in which case i must win because it wins all pairwise competitions. Therefore the the winner of the pairwise competitions inevitably wins the mixed competition.

However, the pairwise champion i may still lose transiently, i.e., $\sigma_{ik}^{\text{mixed}} < 0$ for some other intermediate strain $k \neq j$. For example, this occurs in Supplementary Fig. 4, where the green strain beats both blue and red in pairwise competitions (Supplementary Fig. 4a) but loses transiently to red in the mixed competition at early times (Supplementary Fig. 4b). This effect is due to a higher-order modification to the selection coefficient from the lag-growth coupling term $s_{ijk}^{\text{coupling}}$ (Eq. 3). However, it only persists until the worst remaining strain (blue) effectively goes extinct, after which green then beats red.

SUPPLEMENTARY NOTE 7. TRAIT CONSTRAINTS FOR NON-TRANSITIVE COMPETITIONS

Consider a set of three strains: blue, orange, and green. For competitions starting from equal densities to be non-transitive, the pairwise selection coefficients must satisfy $s_{\text{orange,blue}} > 0$, $s_{\text{green,orange}} > 0$, and $s_{\text{blue,green}} > 0$, which simplify to

$$\begin{aligned} \Delta\tau_{\text{orange,blue}} \log\left(\rho \bar{Y}_{\text{orange,blue}}^{\text{equal}}\right) + \Delta\lambda_{\text{orange,blue}} < 0, \\ \Delta\tau_{\text{green,orange}} \log\left(\rho \bar{Y}_{\text{green,orange}}^{\text{equal}}\right) + \Delta\lambda_{\text{green,orange}} < 0, \\ \Delta\tau_{\text{blue,green}} \log\left(\rho \bar{Y}_{\text{blue,green}}^{\text{equal}}\right) + \Delta\lambda_{\text{blue,green}} < 0, \end{aligned} \quad (36)$$

where $\bar{Y}_{ij}^{\text{equal}} = \left(Y_i^{-1}/2 + Y_j^{-1}/2\right)^{-1}$ is the harmonic mean of Y_i and Y_j with equal densities (cf. Eq. 4). In the top panel of Supplementary Fig. 5a, these three inequalities are represented by the violet, red, and cyan lines, respectively. These inequalities are satisfied by the blue, orange, and green strains as shown here and demonstrated by the competitions in Fig. 4c.

For invasions (where one strain starts at low density and ultimately fixes) to be non-transitive, each strain must beat another strain not just at equal densities, but at all densities. This results in the following inequalities:

$$\Delta\lambda_{\text{orange,blue}} < \begin{cases} -\Delta\tau_{\text{orange,blue}} \log\left(\rho \min\left[Y_{\text{blue}}, Y_{\text{orange}}\right]\right) & \text{for } \Delta\tau_{\text{orange,blue}} < 0 \\ -\Delta\tau_{\text{orange,blue}} \log\left(\rho \max\left[Y_{\text{blue}}, Y_{\text{orange}}\right]\right) & \text{for } \Delta\tau_{\text{orange,blue}} > 0 \end{cases} \tag{37a}$$

$$\Delta\lambda_{\text{green,orange}} < \begin{cases} -\Delta\tau_{\text{green,orange}} \log\left(\rho \min\left[Y_{\text{orange}}, Y_{\text{green}}\right]\right) & \text{for } \Delta\tau_{\text{green,orange}} < 0 \\ -\Delta\tau_{\text{green,orange}} \log\left(\rho \max\left[Y_{\text{orange}}, Y_{\text{green}}\right]\right) & \text{for } \Delta\tau_{\text{green,orange}} > 0 \end{cases} \tag{37b}$$

$$\Delta\lambda_{\text{blue,green}} < \begin{cases} -\Delta\tau_{\text{blue,green}} \log\left(\rho \min\left[Y_{\text{blue}}, Y_{\text{green}}\right]\right) & \text{for } \Delta\tau_{\text{blue,green}} < 0 \\ -\Delta\tau_{\text{blue,green}} \log\left(\rho \max\left[Y_{\text{blue}}, Y_{\text{green}}\right]\right) & \text{for } \Delta\tau_{\text{blue,green}} > 0. \end{cases} \tag{37c}$$

These three inequalities define the violet, red, and cyan lines, respectively, in the bottom panel of Supplementary Fig. 5a. However, the green traits cannot simultaneously satisfy both Supplementary Eqs. 37b and 37c, which we can prove by geometrically showing that the red and cyan lines can never intersect. Without loss of generality we assume the blue strain has the smallest yield ($Y_{\text{blue}} < Y_{\text{orange}}, Y_{\text{green}}$). Now first consider the case where $Y_{\text{orange}} < Y_{\text{green}}$. Then the left branch of the red line has slope $-\log\left(\rho Y_{\text{orange}}\right)$, while the left branch of the cyan line has the steeper slope $-\log\left(\rho Y_{\text{green}}\right)$. Thus the lines diverge in this direction. They also diverge to the right, since the right branch of the red line has slope $-\log\left(\rho Y_{\text{green}}\right)$, which is steeper than the cyan line's slope of $-\log\left(\rho Y_{\text{blue}}\right)$. Since the red line is also constrained to be below the violet line at $\tau = 0$ (by the constraints on the orange strain, Supplementary Eq. 37a), the red and cyan lines therefore never intersect. A similar argument holds if we flip the ordering of the orange and green yields, so that $Y_{\text{green}} < Y_{\text{orange}}$. Therefore it is not possible for three strains to invade each other non-transitively.

However, it is not necessarily true that one strain must always be able to invade all others. For example, Supplementary Fig. 5b shows all invasion competitions for the same three strains in Fig. 4c,d, where they are non-transitive in equal competitions. In this case orange can invade blue, but orange cannot invade green, and green cannot invade either blue or orange. This is because both the blue-green and orange-green pairs are bistable, with an unstable fixed point at some intermediate density.

SUPPLEMENTARY NOTE 8. ADDITIONAL PARAMETER VALUES FOR FIGURES

Figure 3. Lag and growth times are shown in panel (**a**); yields are $Y_{\text{blue}} = 500$, $Y_{\text{red}} = 600$, $Y_{\text{green}} = 750$, and $Y_{\text{orange}} = 1000$ in all panels. In panel (**b**), the three values of ρ are 0.75, 1.5, and 2.25. In panel (**c**), $\rho = 1.32$.

Figure 4. (**a**, **b**) Growth times are $\tau_{\text{blue}} = 1$, $\tau_{\text{orange}} = 0.978$, and $\tau_{\text{green}} = 1.025$; lag times are $\lambda_{\text{blue}} = 0.15$, $\lambda_{\text{orange}} = 0.28$, and $\lambda_{\text{green}} = 0$; and yields are $\rho Y_{\text{blue}} = \rho Y_{\text{orange}} = 10^3$ and $\rho Y_{\text{green}} = 200$. (**c**, **d**) Growth times are $\tau_{\text{blue}} = 1$, $\tau_{\text{orange}} = 1.1$, and $\tau_{\text{green}} = 0.8$; lag times are $\lambda_{\text{blue}} = 1$, $\lambda_{\text{orange}} = 0$, and $\lambda_{\text{green}} = 2.2$; yields are $\rho Y_{\text{blue}} = 10^2$, $\rho Y_{\text{orange}} = 10^3$, and $\rho Y_{\text{green}} = 10^4$.

Supplementary Figure 1. The lag-growth tradeoff is $c = \log 1000$, and the yields are $Y_1 = 500$, $Y_2 = 600$, $Y_3 = 750$, and $Y_4 = 1000$.

Supplementary Figure 2: (**a**) Growth times are $\tau_{\text{blue}} = 1$, $\tau_{\text{red}} = 1.01$, and $\tau_{\text{green}} = 1.02$. (**b**) Growth times are $\tau_{\text{blue}} = 1.02$, $\tau_{\text{red}} = 1.01$, and $\tau_{\text{green}} = 1$. (**c**) Growth times are $\tau_{\text{blue}} = 1.01$, $\tau_{\text{red}} = 1.02$, and $\tau_{\text{green}} = 1$. In all panels the lag-growth tradeoff (which defines the lag times from the growth times via Eq. 6) is $c = \log(2^{1/4} \times 10^3)$, and the yields are $\rho Y_{\text{blue}} = 10^3$, $\rho Y_{\text{red}} = 2^{1/2} \times 10^3$, and $\rho Y_{\text{green}} = 2 \times 10^3$.

Supplementary Figure 3: We sample the yields $\{Y_k\}$ from a Gaussian distribution with mean 10^3 and

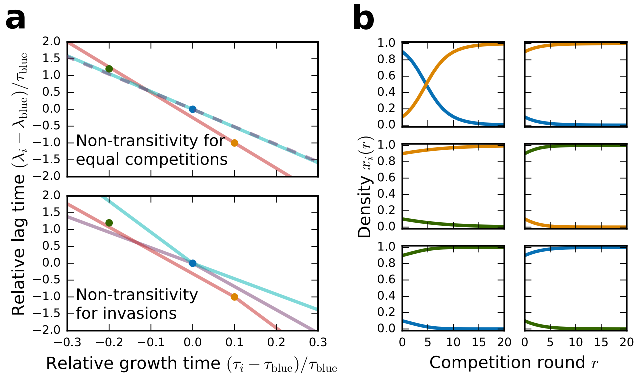

SUPPLEMENTARY FIGURE 5. **Invasions cannot be non-transitive.** (**a**) Diagrams of lag-growth trait space for the same three strains (blue, orange, green) as in Fig. 4c,d. The dots mark the same three strains in both top and bottom panels. For non-transitivity to occur, the orange strain must lie below the violet line (so that it beats the blue strain) and the green strain must lie both below the red line (so that it beats the orange strain) and above the cyan line (so that it loses to the blue strain). The top panel shows these constraints for competitions starting at equal densities (Fig. 4c, Supplementary Eq. 36), while the bottom panel shows these constraints for invasions (Supplementary Eq. 37). Note the violet and cyan lines practically overlap in the top panel, since their slopes $-\log\left(\rho\bar{Y}^{\text{equal}}_{\text{orange,blue}}\right)$ and $-\log\left(\rho\bar{Y}^{\text{equal}}_{\text{blue,green}}\right)$ (Supplementary Eq. 36) are nearly equal because the blue strain has the lowest yield and $\bar{Y}$ is a harmonic mean (Eq. 4). (**b**) Invasion competitions for each pair of strains from (**a**). See Supplementary Note 8 for parameter values.

standard deviation 10^2. We sample growth times $\{\tau_k\}$ from a Gaussian with mean 1 and standard deviation 10^{-2}; we then generate correlated lag times $\{\lambda_k\}$ from the growth times using a "true" correlation coefficient uniformly sampled between -1 and 0. The resource density is $\rho = 1$.

Supplementary Figure 4: Growth times are $\tau_{\text{blue}} = 1$, $\tau_{\text{red}} = 1.01$, and $\tau_{\text{green}} = 2$; lag times are $\lambda_{\text{blue}} = 6.9195$, $\lambda_{\text{red}} = 6.8395$, $\lambda_{\text{green}} = 0$; yields are $\rho Y_{\text{blue}} = \rho Y_{\text{red}} = \rho Y_{\text{green}} = 10^3$.

SUPPLEMENTARY REFERENCES

[1] M. Manhart, B. V. Adkar, and E. I. Shakhnovich. Trade-offs between microbial growth phases lead to frequency-dependent and non-transitive selection. *Proc R Soc B*, 285:20172459, 2018.

[2] S. Pressé, K. Ghosh, J. Lee, and K. A. Dill. Principles of maximum entropy and maximum caliber in statistical physics. *Rev Mod Phys*, 85:1115–1141, 2013.
